# A brain-targeting lipidated peptide for neutralizing RNA-mediated toxicity in Polyglutamine Diseases

**DOI:** 10.1038/s41598-017-11695-y

**Published:** 2017-09-21

**Authors:** Qian Zhang, Mengbi Yang, Kasper K. Sørensen, Charlotte S. Madsen, Josephine T. Boesen, Ying An, Shao Hong Peng, Yuming Wei, Qianwen Wang, Knud J. Jensen, Zhong Zuo, Ho Yin Edwin Chan, Jacky Chi Ki Ngo

**Affiliations:** 1 School of Life Sciences, The Chinese University of Hong Kong, Shatin, Hong Kong SAR China; 2 School of Pharmacy, The Chinese University of Hong Kong, Shatin, Hong Kong SAR China; 3 Department of Chemistry, University of Copenhagen, Thorvaldsensvej 40, 1871 Frederiksberg, Denmark; 4 Gerald Choa Neuroscience Centre, The Chinese University of Hong Kong, Shatin, Hong Kong SAR China

## Abstract

Polyglutamine (PolyQ) diseases are progressive neurodegenerative disorders caused by both protein- and RNA-mediated toxicities. We previously showed that a peptidyl inhibitor, P3, which binds directly to expanded *CAG* RNA can inhibit RNA-induced nucleolar stress and suppress RNA-induced neurotoxicity. Here we report a N-acetylated and C-amidated derivative of P3, P3V8, that showed a more than 20-fold increase in its affinity for expanded *CAG* RNA. The P3V8 peptide also more potently alleviated expanded RNA-induced cytotoxicity *in vitro*, and suppressed polyQ neurodegeneration in *Drosophila* with no observed toxic effects. Further N-palmitoylation of P3V8 (L1P3V8) not only significantly improved its cellular uptake and stability, but also facilitated its systemic exposure and brain uptake in rats via intranasal administration. Our findings demonstrate that concomitant N-acetylation, C-amidation and palmitoylation of P3 significantly improve both its bioactivity and pharmacological profile. L1P3V8 possesses drug/lead-like properties that can be further developed into a lead inhibitor for the treatment of polyQ diseases.

## Introduction

Polyglutamine (PolyQ) diseases, including Machado-Joseph Disease (MJD) and Huntington’s disease, are a group of dominantly inherited progressive neurodegenerative diseases characterized by the existence of expanded CAG trinucleotide repeats within the coding region of disease genes^1^. Through transcription and translation, disease proteins carrying an extended polyQ stretch are detected in the affected tissues. It is widely accepted that polyQ disease toxicity is ascribed to the misfolding and aggregation of disease proteins^2–4^. However, an accumulation of evidence demonstrates that the expanded CAG-repeat RNA also contributes to toxicity in polyQ diseases^5–8^. Various expanded *CAG* RNA pathogenic pathways have recently been described, including the recruitment of muscleblind-like (MBNL) proteins to expanded CAG-repeat RNA foci^9,10^, the generation of small *CAG* RNAs via Dicer cleavage^11,12^, and the activation of nucleolar stress^13,14^.

We previously demonstrated that expanded *CAG* RNA triggers nucleolar stress and eventually induces toxicity both *in vitro* and *in vivo* ^13,14^. Nucleolar stress is a cellular response to the failure in ribosome biogenesis and/or ribosome malfunction^15^. A reduction in ribosomal RNA (*rRNA*) transcription causes an imbalance in the intracellular levels of ribosomal *RNA*s and ribosomal proteins, which triggers ribosome assembly defects and eventually leads to nucleolar stress-induced apoptosis^16,17^. Our previous investigation showed that expanded *CAG* RNA physically interacts with the nucleolin (NCL) protein^13^, a multifunctional nucleolar protein that plays critical roles in precursor *rRNA* (*pre-rRNA*) transcription^18^, processing^19^ and pre-ribosome assembly^18,20^. This RNA-protein interaction leads to *upstream control element* (*UCE*) hypermethylation and the down-regulation of *rRNA* transcription, which induces nucleolar stress^13^. We developed a 13-amino acid peptide inhibitor, P3, which can inhibit NCL-expanded *CAG* RNA interaction and suppress RNA toxicity in polyQ diseases^21^. The P3 peptide was designed based on the structure of the RRM2 domain of NCL^21^ which contains the ribonucleoprotein domain-1 (RNP-1) motif^22^. Our study showed that P3 could directly and preferentially bind to expanded continuous CAG-repeat RNA *in vitro* ^21^. This P3-*CAG* RNA interaction titrated endogenous NCL away from binding to the toxic RNA and restored NCL-*UCE* interaction and *pre-45s rRNA* transcription^21^. Treatment of P3 suppressed expanded *CAG* RNA-induced cell death in a mammalian cell model and neurodegeneration in a *Drosophila* disease model^21^. The calculated maximal inhibitory concentration (IC_50_) of P3 in inhibiting cell death was 4.369 ± 1.140 μM^21^. In this study, we engineered peptide P3 and identified a more potent inhibitor for targeting RNA toxicity in polyQ diseases.

## Results

### Alanine scanning of P3

P3 is derived from the primary structure of NCL^21^. We previously synthesized a series of P3 analogues, in which basic or aromatic residues were substituted with Ala and their binding to expanded *CAG* RNA was measured to identify the amino acid side chains involved in the interaction with expanded *CAG* RNA. Our results showed that Lys3, Lys5, Tyr9, and Phe12 are indispensable and serve as the pharmacophores for RNA binding^21^. To further illustrate how the mutations affect the interaction between P3 and RNA, we performed isothermal titration calorimetry (ITC) experiments to characterize the interaction between each individual mutant (Supplementary Table S1) and expanded *CAG* RNA. All ITC experiments were performed using high ratios of peptide-to-RNA concentrations to increase the experiment’s sensitivity. The heat generated by peptide dilution was determined by titrating the peptides into buffer only and was then subtracted from the binding titration curves of the corresponding peptides. The determined equilibrium dissociation constant K_D_ of P3 is ~8.4 µM with both favorable enthalpy (ΔH = −4.5 kcal/mol) and entropy (TΔS = 2.4 kcal/mol), suggesting that the peptide-RNA interaction is energetically driven by both favorable enthalpic and entropic components.

On the other hand, the determined equilibrium dissociation constant K_D_ of P3MT3 and P3MT4 revealed that the mutations of the aromatic residues reduced the binding affinity to 16 and 17 μM respectively, whereas mutations of the lysines (P3MT1, P3MT2 and P3MT5) had a more dramatic effect and reduced the affinity by ~3–12 fold (Supplementary Table S1). In particular, the K_D_ of P3MT2 and P3MT5 were increased to 100 µM and 51 µM respectively, indicating that among all the identified pharmacophores, Lys5 and Lys13 are most critical for RNA binding.

### Amino acid substitutions of P3

The observation that favorable entropic change contributes to P3 interaction with expanded *CAG* RNA suggests that the peptide may have undergone conformational changes upon binding to RNA. Structure prediction of the P3 peptide using the PEP-FOLD server^23^ suggested that although P3 does not adopt a well-defined tertiary structure, it may preferentially adopt a loosely folded coil conformation that positions the pharmacophores Lys3, Tyr9, and Phe12 on the same surface of the peptide, which may in turn facilitate the recognition and interaction of RNA (Supplementary Fig. S1). Such conformation is maintained partially by a network of interaction mediated by the side chain of Asp1. To test whether the orientation of Asp1 is important, we restricted it by substituting Gly2 with D-alanine (P3V1). Our result shows that the binding affinity of P3V1 was improved by 4-fold which gave a K_D_ of 2 µM. Such improvement could be due to better stabilization of the RNA-interacting surface via the restriction of Asp1 or because the replacement of Gly by D-Ala minimized the entropic loss upon binding by restricting the flexibility of the peptide. Analysis of the ITC results reveals that the change in the Gibbs free energy of P3V1 mainly originated from a favorable gain in enthalpy, indicating that the former scenario is more likely. Considering that both Phe12 and Lys13 are important for RNA binding, we kept the 13-mer P3 as our minimal construct for further studies.

To improve the binding affinity and specificity of P3 toward expanded *CAG* RNA, we performed amino acid substitutions at individual pharmacophores using natural amino acids with similar properties, i.e. Tyr and Phe to other aromatic side chains; Lys to Arg (Table 1). Substitution of Tyr9 with the larger Trp side chain (P3V2) resulted in a lower K_D_ of 2.2 µM and the substitution of Phe with less hydrophobic Tyr (P3V3) and Trp (P3V4) also improved the binding of peptide to expanded *CAG* RNA by nearly 2- to 4-fold, respectively. These results indicate that although alterations of the size or polarity of the aromatic residues improved the binding affinity of P3, only modest improvements were obtained. On the other hand, the K_D_ value of P3V5, in which Lys3 and Lys5 both mutated to Arg residues, was approximately 0.86 µM, reflecting a nearly 10-fold improvement over the P3 peptide. Contrarily, when we mutated all three Lys to Arg (P3V6), the K_D_ of the peptide increased to 23 µM, indicating that the replacement of the amine of Lys13 with a guanidinium is not favored.

**Table 1:** Binding affinity of P3V1-9 expanded *MJD*_*CAG78*_ RNA.

Peptide	Sequence	K_D_ (μM)
P3WT	Asp-Gly-Lys-Ser-Lys-Gly-Ile-Ala-Tyr-Ile-Glu-Phe-Lys	8.37 ± 3.83
P3 variant 1 (P3V1)	Asp-{d-Ala}-Lys-Ser-Lys-Gly-Ile-Ala-Tyr-Ile-Glu-Phe-Lys	2.00 ± 0.34
P3 variant 2 (P3V2)	Asp-Gly-Lys-Ser-Lys-Gly-Ile-Ala-Trp-Ile-Glu-Phe-Lys	2.21 ± 1.27
P3 variant 3 (P3V3)	Asp-Gly-Lys-Ser-Lys-Gly-Ile-Ala-Tyr-Ile-Glu-Tyr-Lys	1.74 ± 0.67
P3 variant 4 (P3V4)	Asp-Gly-Lys-Ser-Lys-Gly-Ile-Ala-Tyr-Ile-Glu-Trp-Lys	4.44 ± 0.47
P3 variant 5 (P3V5)	Asp-Gly-Arg-Ser-Arg-Gly-Ile-Ala-Tyr-Ile-Glu-Phe-Lys	0.86 ± 0.17
P3 variant 6 (P3V6)	Asp-Gly-Arg-Ser-Arg-Gly-Ile-Ala-Tyr-Ile-Glu-Phe-Arg	23.78 ± 2.78
P3 variant 7 (P3V7)	Asp-Gly-Lys-Ser-Lys-Gly-Ile-Ala-Tyr-Ile-Glu-Phe-Lys-NH_2_	3.67 ± 0.26
P3 variant 8 (P3V8)	Ac-Asp-Gly-Lys-Ser-Lys-Gly-Ile-Ala-Tyr-Ile-Glu-Phe-Lys-NH_2_	0.33 ± 0.04
P3 variant 9 (P3V9)	Ac-Asp-Gly-Arg-Ser-Arg-Gly-Ile-Ala-Tyr-Ile-Glu-Phe-Lys-NH_2_	2.34 ± 0.49

P3WT or P3 variants (0.7 mM) were titrated into *MJD*_*CAG78*_ RNA (0.5 µM) and the thermal titration data were fitted to the ‘one binding site model’ to determine the dissociation constant (K_D_). WT indicates wild type. Data are expressed as mean ± S.E.M. for at least 3 independent experiments.

### Termini modifications improve P3 activity

Next, we explored whether modifications at the N- and C-termini could improve the binding and inhibitory activity of P3. N-terminal acetylation and C-terminal amidation neutralize the charges at both ends of the peptide and we speculated that it may increase its biological activity. The C-terminal amide was incorporated by solid-phase synthesis of the peptide on a Rink amide linker, while the N-terminal modifications were introduced after assembly of the peptide and before acidolytic release of the completed structure. The K_D_ value of P3V7 was determined to be 2 µM using ITC, indicating that C-terminal amidation improved the peptide affinity to expanded RNA. When the N-terminus was also acetylated, the dissociation constant of P3V8 was further lowered to 0.33 µM (Table 1). Based on these observations, we further synthesized P3V9, which combined the modifications of P3V8 and P3V5 (N-acylation, C-amidation and Lys-to-Arg mutation) with the hope to further improving the binding affinity of the peptide. To our surprise, the binding affinity of the new peptide was not improved, as the K_D_ value was 2.3 µM (Table 1). We next investigated whether substitution of Lys3 and Lys5 with other non-natural Lys and Arg analogs would improve the activity of P3V8. The homologated Arg analog, homoarginine (hArg), and two shortened Lys analogs, ornithine (Orn) and 2,3-diaminopropionic acid (Dap), were used for substitution in our studies (Supplementary Table S2, P3 variants 14–19 respectively). Our results reveal that when either Lys3 or Lys5 was replaced by any of the amino acid analogs, no binding could be detected between the P3 variant and the expanded *CAG* RNA using ITC. This indicates that the chain length of Lys in P3V8 is crucial for its bioactivity. In addition, the linear dimer of P3V8, which has two copies of the peptide in close vicinity, did not show any binding (Supplementary Table S2, P3V20 and P3V21). The abolishment of the bioactivity may be due to steric hindrance.

To test whether P3V8 specifically targets expanded *CAG* RNA, ITC experiments were performed using unexpanded *CAG* RNA (*MJD*_*CAG27*_ RNA), expanded *CAG* RNA (*MJD*_*CAG78*_ RNA), and expanded *CAA/G* RNA *(MJD*_*CAA/G78*_ RNA), respectively, to investigate whether the binding of P3V8 is RNA expansion-dependent and sequence-dependent (Supplementary Table S3). Our results demonstrate that P3V8 bound unexpanded RNA or expanded *CAA/G* RNA with nearly 6–9-fold lower affinity, which indicates that P3V8 is expanded *CAG* RNA-specific.

### P3 derivatives showed improved suppressive effects on polyglutamine neurodegeneration *in vivo*

**Figure 1:**
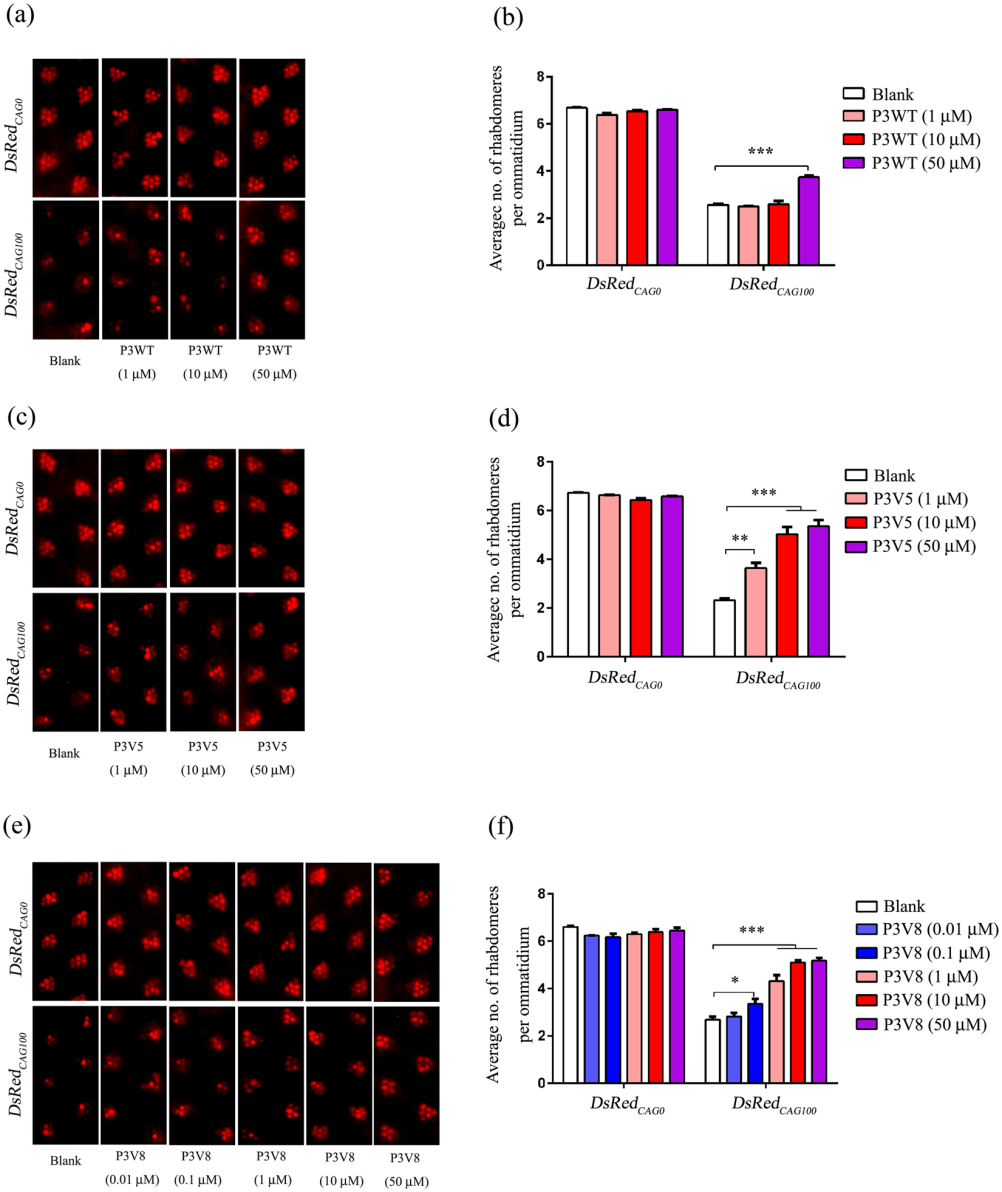
P3V8 most effectively suppresses expanded *CAG* RNA-induced RNA toxicity *in vivo*. (**a**) Effect of P3WT on suppressing *DsRed*_*CAG100*_ neurodegeneration in *Drosophila*. (**b**) Statistical analysis of panel (a). (**c**) Effect of P3V5 on suppressing *DsRed*_*CAG100*_ neurodegeneration in *Drosophila*. (**d**) Statistical analysis of panel (c). (**e**) Effect of P3V8 on suppressing *DsRed*_*CAG100*_ neurodegeneration in *Drosophila*. (**f**) Statistical analysis of panel (e). Pseudopupil assay was performed on 12 day-old adult flies. The flies were of genotypes *w*; *gmr-GAL4 UAS-DsRed*_*CAG0*_/+; +/+ and *w*; *gmr-GAL4*/+; *UAS-DsRed*_*CAG100*_/+. Data are expressed as mean ± S.E.M. for at least 3 independent experiments. *Indicates *P* < 0.05, **indicates *P* < 0.01 and ***indicates *P* < 0.001.

Given that P3V5 and P3V8 bind expanded *CAG* RNA with significantly higher affinities than P3, we next tested their inhibitory activities against polyQ neurodegeneration *in vivo*. We previously demonstrated that the full-length *MJD*_*CAG*_ transgenic *Drosophila* model, *flMJD*_*CAG27/84*_, can be used to investigate the suppression effects of synthetic peptide inhibitors on both expanded RNA and polyQ protein toxicities^21^. Here we utilized the *DsRed*_*CAG0/*100_ *Drosophila* model to investigate the suppressive effects of the P3 analogs on RNA toxicity. In this model, the CAG repeat is located in the 3′ untranslated region of the *DsRed* reporter gene^7^. The transcribed expanded *CAG* RNA is not translated into polyQ protein, and thus the toxicity is solely attributed to the expression of *DsRed*_*CAG100*_ RNA. In brief, the expression of expanded *CAG* RNA causes severe retinal degeneration in the animal that can be quantified by a pseudopupil assay^7^. Inhibitors that can suppress neurotoxicity will result in the recovery of the number of rhabdomeres within the ommatidium. To test our derivatives, third instar larvae were fed different amounts of peptide dissolved in sucrose solution for 2 hr and then allowed to culture in standard fly food until the flies were 12 days old, at which time they were sacrificed for the pseudopupil assay. We observed that treatment with 50 µM of P3 peptide moderately suppressed neurotoxicity in *DsRed*_*CAG100*_ flies (pseudopupil score, 3.74 ± 0.05; Fig. 1a and b). In contrast, derivatives P3V5 and P3V8 both significantly suppressed neurotoxicity and recovered the number of rhabdomeres with much greater efficiency (Fig. 1c–f). In particular, P3V8 displayed a high level of potency against the neurotoxicity in *DsRed*_*CAG100*_ flies and achieved a pseudopupil score of 4.32 ± 0.25 in comparison to P3V5’s score of 3.64 ± 0.22 after 1 µM treatment (Fig. 1d and f). Based on the results from the ITC experiments and pseudopupil assay, the peptide derivative P3V8 seems to be the most potent P3 variant that exerts inhibitory activity against expanded *CAG* RNA-induced neurotoxicity.

### P3V8 restored the expression level of *pre-rRNA* in a fly model expressing expanded *CAG* RNA

**Figure 2:**
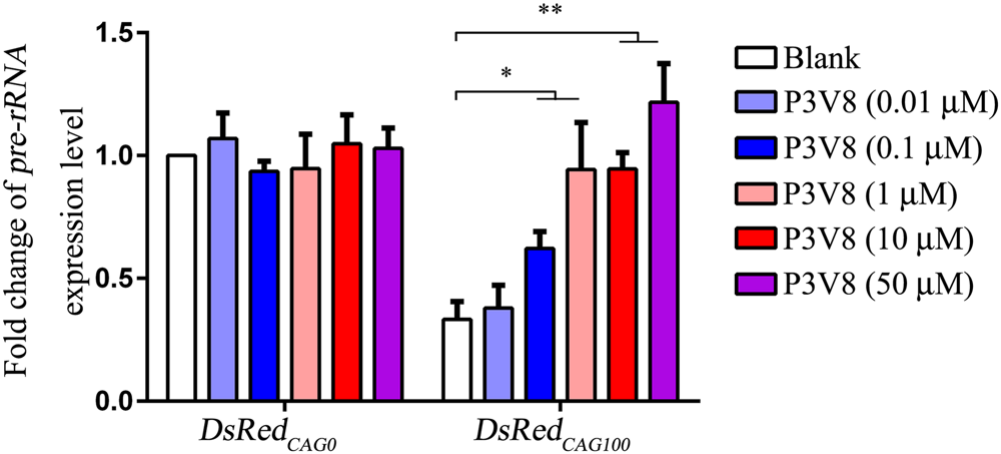
Treatment of P3V8 peptide suppresses nucleolar stress *in vivo*. Treatment of P3V8 restored *pre-rRNA* levels in *DsRed*_*CAG100*_ flies. Real-time PCR was performed to determine the levels of *pre-rRNA*. Data are presented as fold change of the relative *pre-rRNA* expression levels compared with the blank. Experiments were repeated at least 3 times, and data are expressed as mean ± S.E.M. *Indicates *P* < 0.05 and **indicates *P* < 0.01.

*Ribosomal RNA* (*rRNA*) synthesis occurs within the nucleolus^15^. We previously showed that expanded *CAG* RNA induces nucleolar stress by preventing the nucleolar protein NCL from binding to the upstream control element of the *rRNA* promoter, leading to the downregulation of *rRNA* transcription^13^. To investigate whether P3V8 suppressed neurotoxicity by alleviating expanded *CAG* RNA-induced nucleolar stress *in vivo*, we measured the expression levels of *pre-rRNA* in *DsRed*_*CAG100*_ flies treated with the peptide. Real-time PCR analysis showed that P3V8 could restore the transcript level of *pre-rRNA* in a dose-dependent manner. When *DsRed*_*CAG100*_ flies were treated with 1 μM of P3V8, the *pre-rRNA* level was fully recovered to the *DsRed*_*CAG0*_ control level (Fig. 2). This confirms that P3V8 subdued polyQ neurodegeneration *in vivo* by effective mitigation of the expanded *CAG* RNA-induced nucleolar stress.

### P3V8 suppressed expanded *CAG* RNA-induced cell death by modulating *rRNA* transcription *in vitro*

**Figure 3:**
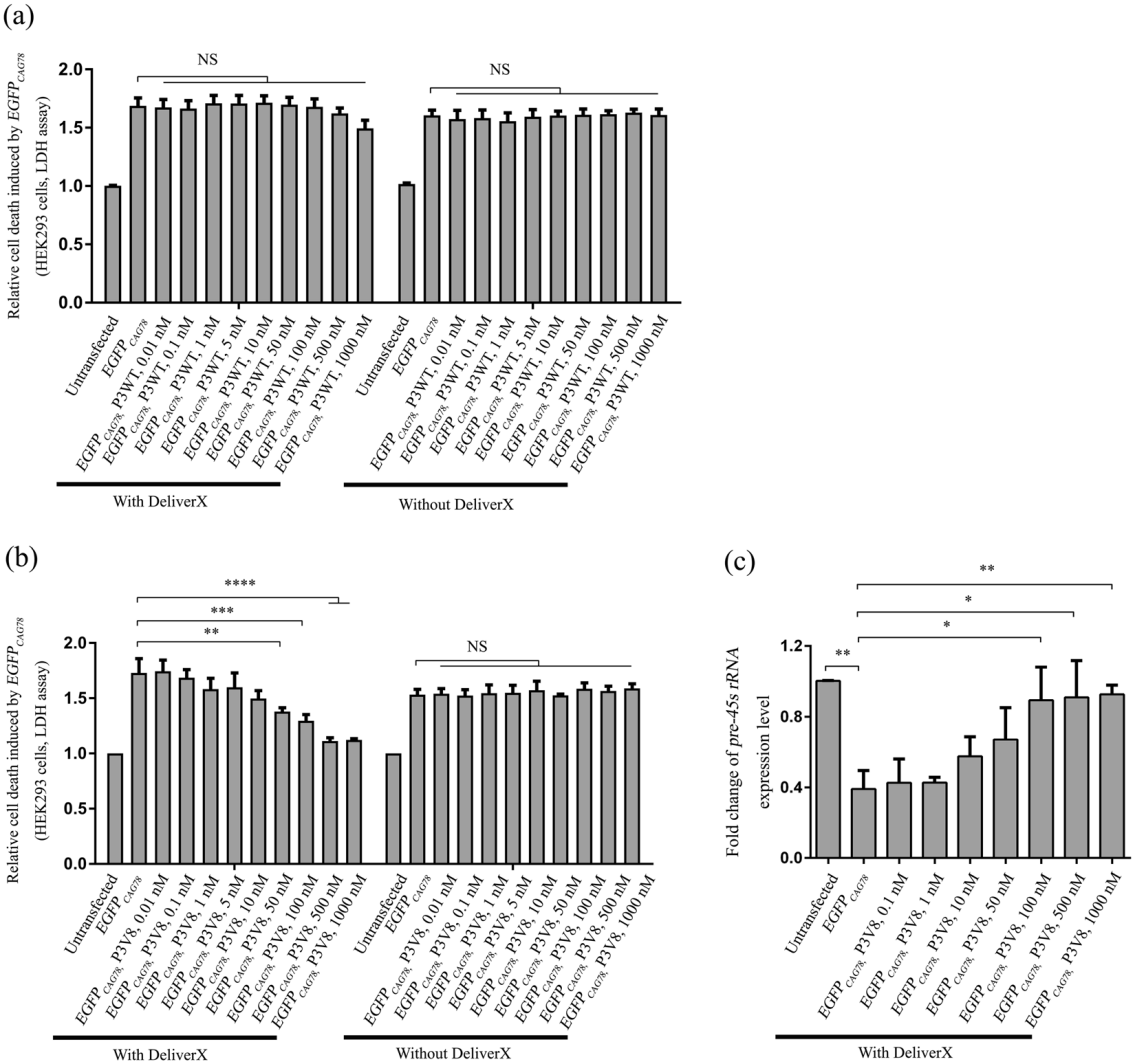
P3V8 effectively inhibits cytotoxicity induced by expanded *CAG* RNA *in vitro*. (**a**) Dose-dependent effect of synthetic P3WT in the absence or presence of DeliverX on the inhibition of cell death in *EGFP*_*CAG78*_ RNA-expressing HEK293 cells. (**b**) Dose-dependent effect of synthetic P3V8 in the absence or presence of DeliverX on the inhibition of cell death in *EGFP*_*CAG78*_ RNA-expressing HEK293 cells. Left of the charts: various amount of P3WT (**a**) or P3V8 (**b**) were transfected into individual culture wells by DeliverX 4 hr after plasmid transfection. Right of the charts: P3WT (**a**) or P3V8 (**b**) was added into individual culture wells immediately after plasmid transfection. A lactate dehydrogenase (LDH) cytotoxicity assay was performed. (**c**) Treatment of P3V8 (with DeliverX) restored *pre-45s rRNA* levels in *EGFP*_*CAG78*_ RNA-expressing HEK293 cells. Cells were transfected with various amount of P3V8 using DeliverX. Data are presented as fold change of the relative *pre-45s rRNA* expression levels compared with the untransfected samples. Experiments were repeated at least 3 times and data are expressed as mean ± S.E.M. NS indicates no significance. *Indicates *P* < 0.05, **indicates *P* < 0.01, ***indicates *P* < 0.001 and ****indicates *P* < 0.0001.

We next investigated whether P3V8 could also mitigate expanded *CAG* RNA-induced toxicity in mammalian cells. Overexpression of *EGFP*_*CAG78*_ RNA in HEK293 cells has been shown to induce nucleolar stress and apoptosis^21^. To compare the inhibitory activity of P3WT and P3V8, we used the peptide transfection reagent DeliverX (DX) to deliver P3WT and P3V8 to *EGFP*_*CAG78*_ RNA-expressing HEK293 cells. Figure 3a shows that irrespective of the addition of DX transfectant, treatment of P3WT lower than 1 µΜ did not elicit any significant suppression effect on expanded *CAG* RNA-induced cell death. Although P3V8 alone did not exert any cellular effect, its administration with the aid of DX could effectively alleviate the cytotoxicity induced by expanded *CAG* RNA (Fig. 3b). To confirm that P3V8 inhibited expanded *CAG* RNA-mediated nucleolar stress, we measured the levels of *pre-45s rRNA* in *EGFP*_*CAG78*_ RNA-expressing cells with or without treatment of P3V8 (Fig. 3c). Overexpression of the expanded *CAG* RNA reduced the level of *pre-45s rRNA* to approximately 40% of the untransfected control. Upon P3V8 treatment, a dose-dependent restoration of *pre-45s rRNA* level was observed (Fig. 3c). Taken together, our results demonstrate that P3V8 could effectively mitigate expanded *CAG* RNA-induced nucleolar stress and neutralize the cytotoxic effect.

### Assessment of P3V8 toxicity

Because we aim to develop P3V8 into a potent inhibitor that targets RNA toxicity in degenerating cells within the nervous system, we assessed whether P3V8 had deleterious effects on neuronal cells by determining lactate dehydrogenase release in the culturing medium of rat cortical neurons. After incubation of P3V8 in concentrations ranging from 0.01 nM to 1 µM for 72 hr, no significant cell death was observed in the P3V8-treated neurons when compared to the untreated control (Supplementary Fig. S2). It should be noted that the peptide also did not show any observable effect when *DsRed*_*CAG0*_ *Drosophila* was treated with P3V8 at up to 50 µM (Fig. 1e and f). This indicates that P3V8 exhibits low cytotoxicity both *in vitro* and *in vivo*.

### N-terminally acylated P3V8 showed enhanced cellular uptake

**Figure 4:**
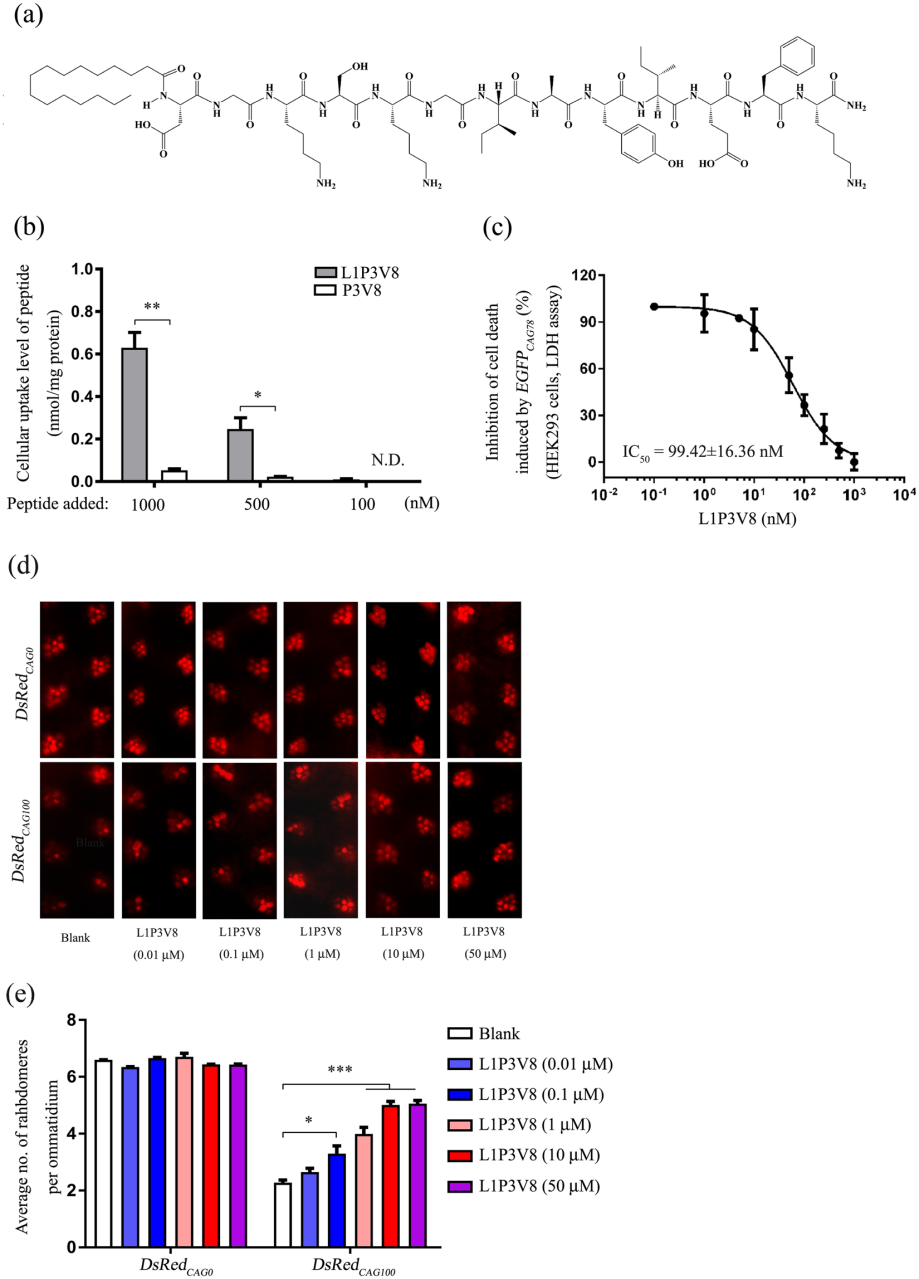
L1P3V8 effectively inhibits neurodegeneration induced by expanded *CAG* RNA *in vitro* and *in vivo*. (**a**) Chemical structure of L1P3V8. (**b**) Comparison of cellular uptake level of P3V8 and L1P3V8 in HEK293 cells. Cellular uptake level was measured 3 hr after 100, 500 or 1000 nM treatment of respective peptide. The total amount of peptide in the cell lysate was normalized to total protein level in cell lysate. (**c**) Dose-dependent effect of synthetic L1P3V8 on the inhibition of cell death in *EGFP*_*CAG78*_ RNA-expressing HEK293 cells. Various amount of L1P3V8 were added into individual culture wells immediately after plasmid transfection. LDH cytotoxicity assay was performed. The IC_50_ value represents the concentration of peptides that reduced LDH enzyme activity by 50% when compared with the no peptide treatment control group. (**c**) Effect of L1P3V8 on suppressing *DsRedCAG*_*100*_ neurodegeneration in *Drosophila*. (**d**) Statistical analysis of panel (c). Pseudopupil assay was performed on 12 day-old adult flies. The flies were of genotypes *w*; *gmr-GAL4 UAS-DsRed*_*CAG0*_/+; +/+ and *w*; *gmr*-*GAL4*/+; *UAS-DsRed*_*CAG100*_/+. Data are expressed as mean ± S.E.M. for at least 3 independent experiments. *Indicates *P* < 0.05 and **indicates *P* < 0.01.

Acylation of peptides with long-chain lipids has been shown to improve their cellular uptake, *in vivo* half-life, and membrane permeability and to change their pharmacokinetic properties^24–26^. Importantly, lipid solubility is a key factor for the transportation of inhibitors across the blood-brain barrier. We therefore investigated whether acylation of P3V8 could improve its cellular uptake *in vitro* as well as bioavailability both *in vitro* and *in vivo*. The L1P3V8 peptide was generated by introducing palmitic acid N-terminally to P3V8 in place of N-acetylation (Fig. 4a). LC/MS was used to detect the level of P3V8 or L1P3V8 uptaken by HEK293 cells after 3 hr treatment. Figure 4b shows that higher cellular concentration of L1P3V8 was measured in HEK293 cells when compared to that of P3V8 in both 500 nM and 1 µM treatment groups. While we could detect a low cellular concentration of L1P3V8 in HEK293 cells treated with 100 nM of peptide, no uptake of P3V8 was detected (Fig. 4b). These data collectively demonstrated that the lipidation of P3V8 improved peptide cellular uptake. We next administered L1P3V8 to *EGFP*_*CAG78*_ RNA-expressing HEK293 cells without any DX peptide transfecting reagents to determine whether lipidation can facilitate the cellular uptake of the peptide. As shown in Fig. 4c, L1P3V8 alone effectively inhibited expanded *CAG* RNA-induced cell death with an empirical IC_50_ value of ~100 nM. In addition, L1P3V8 showed no cytotoxic effects on rat cortical neurons and *DsRed*_*CAG0*_ *Drosophila* model, and no obvious alteration in egg-to-adult viability of wild type flies (Supplementary Figs S3 and S4 and Fig. 4d). Treatment of *DsRed*_*CAG100*_ *Drosophila* with L1P3V8 also suppressed retinal degeneration (Fig. 4d and e). These findings indicate that lipidation of P3V8 improved its cell penetration properties without affecting its inhibitory activity.

### Lipidation improved P3V8 *in vitro* stability

To test whether lipidation could also improve the stability of P3V8, we compared the stabilities of P3V8 and L1P3V8 in rat plasma and brain homogenate via *in vitro* incubation to estimate their extent of degradation in plasma and brain tissues. The results shown in Table 2 indicate that the stability of L1P3V8 in both rat plasma and rat brain homogenate at 37 °C was significantly improved in comparison to that of P3V8. At the concentration of 2000 ng/mL, only 21% and 3% of P3V8 remained stable in plasma and brain homogenates, respectively, whereas nearly all L1P3V8 remained intact in plasma and around 45% of L1P3V8 could be detected in the brain homogenate after 1 hr of incubation. When the incubation was extended to 3 hr, around 87% and 21% of L1P3V8 were still detectable in the plasma and brain homogenates, respectively, whereas P3V8 was almost completely degraded. Such improved stability of L1P3V8 was also observed in plasma when the experiments were repeated using 1000 ng/mL and 500 ng/mL of L1P3V8. Because the concentrations of both P3V8 and L1P3V8 after incubation at 500 ng/mL with brain homogenates were below the limit of quantitation (LOQ), no conclusion on comparison of their stabilities in brain homogenate could be drawn. Nonetheless, our results demonstrate that lipidation of P3V8 with palmitic acid significantly improved its stability in different biological matrices.

**Table 2:** Stability of P3V8 and L1P3V8 in different biological matrices after incubation at 37 °C.

		Percentage of remained (%)			
		Incubation for 1 hr		Incubation for 3 hr	
	Incubation Conc. (ng/mL)	P3V8	L1P3V8	P3V8	L1P3V8
Plasma	2000	20.7 ± 3.3	98.8 ± 5.0^***^	<0.6^#^	86.9 ± 5.5
	1000	8.6 ± 0.5	98.7 ± 3.9^***^	<1.3^#^	81.2 ± 1.5
	500	<2.5^#^	88.1 ± 4.9	N/A	73.8 ± 5.7
Brain homogenate	2000	3.3 ± 0.6	45.0 ± 3.5^***^	<0.6^#^	21.2 ± 3.0
	1000	4.0 ± 1.6	22.8 ± 1.2^***^	<1.3^#^	15.8 ± 0.7
	500	<2.5^#^	<20^#^	N/A	<20^#^

Data are presented as mean ± S.E.M. for 5 independent experiments. ***Indicates *P* < 0.001, significant difference compared with P3V8. ^#^Indicates the concentration was below the lowest limit of quantification (12.5 ng/mL for P3V8 and 100 ng/mL for L1P3V8). N/A indicates not applicable.

### *In vivo* pharmacokinetic properties and brain uptake of P3V8 and L1P3V8

**Figure 5:**
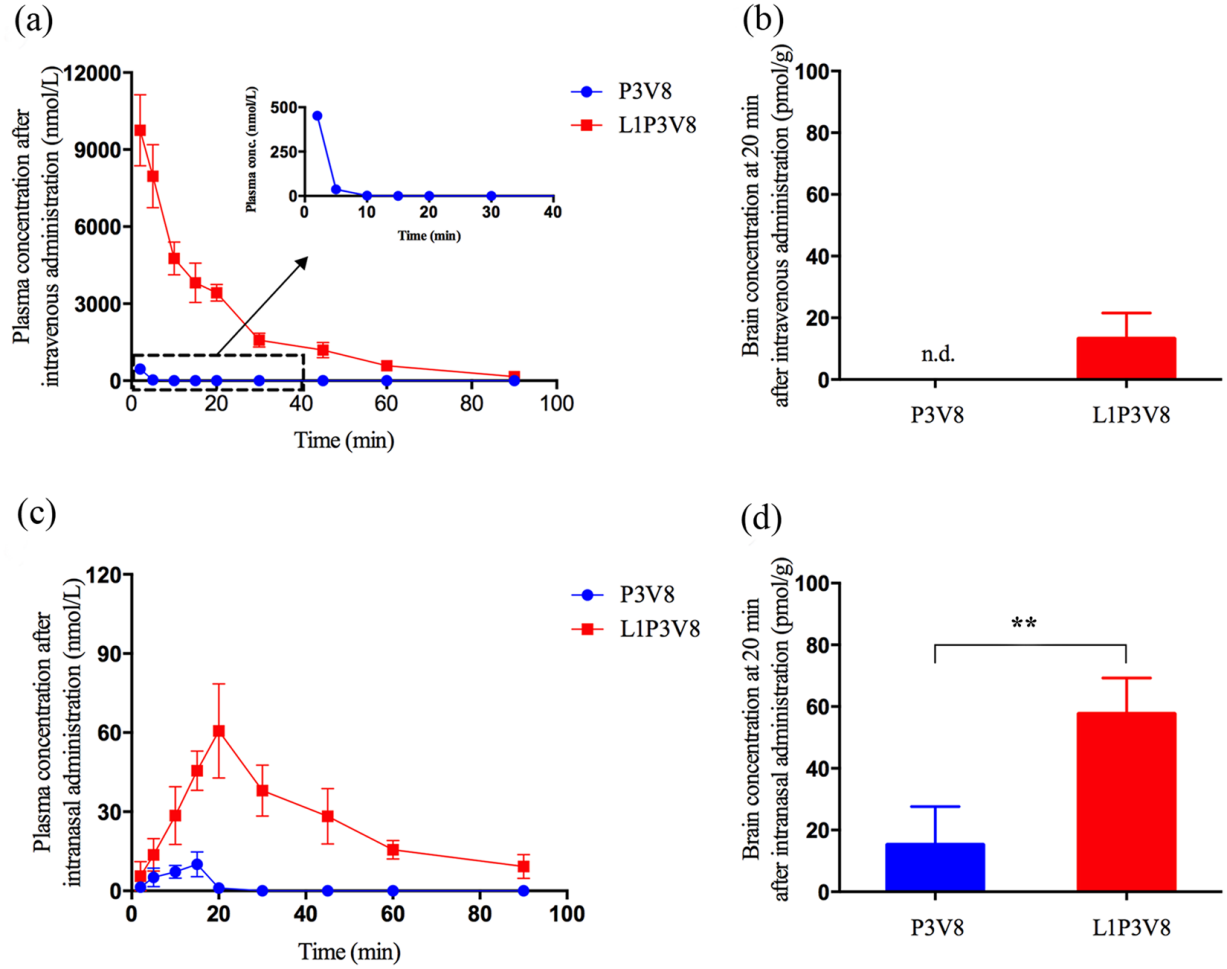
*In vivo* pharmacokinetic study and brain uptake of 3 μmol/kg P3V8 or L1P3V8 in rats. (**a**) Inhibitor plasma concentration-time profiles of P3V8 and L1P3V8 following intravenous administration. (**b**) Brain concentrations of P3V8 (n.d.: not detected) and L1P3V8 at 20 min after intravenous administration (**c**) Inhibitor plasma concentration-time profiles of inhibitors administered via the intranasal route after pre-treatment with 0.5% chitosan. (**d**) Brain concentrations of P3V8 and L1P3V8 at 20 min after intranasal administration. Data are presented as mean ± S.E.M. for 6 independent experiments. **Indicates *P* < 0.01.

The plasma concentration versus time profiles of P3V8 and L1P3V8 after intravenous administration at 3 μmol/kg in Sprague Dawley (SD) rats were studied and compared (Fig. 5a and Table 3). Without lipidation, P3V8 was quickly eliminated in the plasma 10 min after administration and its half-life was too short to be determined. In contrast, L1P3V8 had a significantly longer half-life of 17 min (Table 3) and remained quantifiable at 190 nmol/L in the plasma 90 min after dosing (Fig. 5a). The maximum observed drug concentration (C_max_) and integrated time-concentration responses (area under the curve, AUC) of L1P3V8 were also significantly higher than that of P3V8 (Table 3), indicating better plasma stability after lipidation. The ability of P3V8 and L1P3V8 to be taken up by the brain was also analyzed after intravenous bolus injection. It was noticed that no P3V8 and 13 pmol/g L1P3V8 was detected in the rat brain 20 min after administration (Fig. 5b), suggesting that intravenous administration might not be suitable for brain delivery of these peptides. These undesirable pharmacokinetic properties led us to explore an alternative route of delivery of these peptides to the brain. One of the options is intranasal administration. Intranasal delivery involves the externally exposed olfactory or trigeminal nerve systems and thus is the most direct method of noninvasive delivery method to the brain^27^. Both P3V8 and L1P3V8 were administered intranasally into SD rats at a dose of 3 μmol/kg with pretreatment of 0.5% mucoadhesive chitosan. The pharmacokinetic profiles obtained are significantly different from those obtained after intravenous administration (Fig. 5c and Table 3). It was noted that the plasma levels of the intranasally administered inhibitors were significantly lower than that from intravenous administrations. The concentration of P3V8 in plasma peaked at 15 min and diminished rapidly within 20 min, whereas L1P3V8 concentration peaked at 18 min and remained stay around 9 nmol/mL at 90 min. Similar to the observations from intravenous administration, both C_max_ and AUC of L1P3V8 were significantly higher than those of P3V8 after intranasal administration, which clearly demonstrates again that lipidation of the inhibitor increased its systemic exposure. In addition, significantly higher concentrations of L1P3V8 (58 pmol/g) and P3V8 (15 pmol/g) in the brain were achieved 20 min after intranasal dosing, as compared to those from intravenous administration, indicating better brain uptake via the intranasal route (Fig. 5d). Taken together, our findings support that the combined strategies of lipidation and intranasal administration significantly improve the pharmacokinetic properties and brain uptake of our peptide inhibitor.

**Table 3:** Comparison of pharmacokinetic parameters of P3V8 and L1P3V8 after intravenous or intranasal administrations at 3 μmol/kg.

	Intravenous administration		Intranasal administration (Pre-treated with 0.5% chitosan)	
	P3V8	L1P3V8	P3V8	L1P3V8
C_max_ (nmol/L)	452 ± 22	10394 ± 1225^***^	12 ± 2	74 ± 13^***^
T_max_ (min)	2 ± 0	2 ± 0	15 ± 4	18 ± 1
AUC_0-last_ (min * nmol/L)	1212 ± 80	179470 ± 18874^***^	85 ± 32	2277 ± 558^***^
T_1/2_ (min)	N/A	17 ± 4	N/A	37 ± 5

Data are presented as mean ± S.E.M. for 6 independent experiments. ***Indicates *P* < 0.001, significant difference compared with P3V8. N/A indicates the parameter is not applicable due to insufficient time points with detectable plasma concentrations in elimination phase.

## Discussion

Despite our growing knowledge of the role of RNA transcripts in the pathological mechanisms of polyQ diseases, there is a lack of inhibitors that can counteract their neurotoxicity. On the basis that sequestration of NCL by expanded *CAG* RNA induces cytotoxicity, we recently developed a peptide inhibitor P3 that disrupts NCL-RNA interaction and consequently mitigates RNA toxicity in polyQ diseases. In this study, we determined the equilibrium dissociation constant K_D_ of P3 using ITC and discovered that the binding of P3 to RNA is both enthalpically and entropically favored. Short peptides like the 13-mer P3 usually assume flexible conformations in an aqueous solution and therefore suffer entropic loss upon binding to their targets. The finding that P3 binds expanded RNA with a favorable TΔS suggests that P3 may have undergone a conformation change upon binding to the RNA in solution.

When characterizing the effects of Ala substitutions of the pharmacophores of P3 on its interaction with expanded *CAG* RNA, we found that the basic Lys residues are more important than the aromatic residues in the interaction with expanded RNA. Substitution of any of the three Lys in P3 greatly reduced its binding affinity to RNA, indicating that electrostatic interactions play a dominant role in the affinity of P3. This is no surprise because CAG-repeat RNA duplex adopts an A’ helical conformation that is intermediate to the A- and B-forms of nucleic acids and is highly electronegative on its surface and thus charge-complementary with the key pharmacophores of P3. As described above, the substitution of Lys at positions 3 and 5 by Arg remarkably improved the binding affinity of the peptide by nearly 10-fold (Table 1, P3V5). This observation suggests that the higher pKa of arginine and its guanidinium group may have further strengthened the ionic interaction with the RNA. Surprisingly, when we further mutated Lys13 to arginine, the binding affinity of the peptide was adversely reduced by nearly 3-fold, indicating that the amine group of Lys13, but not the charge alone, is critical for the binding of P3 and RNA. Further research is needed to investigate whether the side chain amine of Lys13 is important for stabilizing the peptide for RNA recognition or for mediating the critical interaction with nucleic acid.

Previous studies have shown that the charge neutralization of the N- and C-termini of synthetic peptides by acetylation and amidation can improve their stabilities in cell cultures and serum, thus improving their biological activity. Based on these findings, we generated P3V8, which is capped by acetylation and amidation at the N- and C-termini, respectively, in an attempt to improve the biological activity of P3 and in hopes that the same strategy can be applied to other P3 derivatives. We observed that capping of both termini of P3 not only improved its binding affinity to expanded *CAG* RNA (K_D_ =0.33 µM) but also significantly improved its potency both *in vivo* and *in vitro*. In a recent study of compounds that block Alzheimer’s Aβ channel activity, Flora and colleagues showed that capping of the amine and carboxyl groups of free histidine helps to improve the residue activity by preventing nonspecific interaction with other reactive residues in the target^28^. We speculate that the capping of P3, now termed P3V8, might have exerted a similar effect and prevented nonspecific interaction between the peptide and the RNA’s negatively charged surface, thus improving the binding and efficacy of the peptide. Furthermore, the slight increase in pI of the peptide might also have contributed to its better affinity to the electrostatically negative surface of RNA (P3V8, pI = 10.02 vs. P3, pI = 9.64).

With the success of P3V8, we applied the same capping strategy to P3V5 to further improve its activity. However, as described above, the combination of Lys-to-Arg mutations of residues 3 and 5 and the capping of the peptide termini did not improve the peptide’s binding affinity but instead reduced the K_D_ to about 2.3 µM. We speculate that upon N- and C-termini modifications, the interactions of the arginines and RNA might have been altered and that P3V9 likely adopts a different binding mode than P3V5 or P3V8. Moreover, the lengths of the basic side chains at residues 3 and 5 also appear to play a determining role in the binding of the peptide, as either lengthening or shortening the basic side chains abolished the interaction between the inhibitors and expanded RNA, suggesting that the distances between the basic side chains and the RNA is critical and that only limited conformational flexibility is allowed after P3V8 binds to the RNA. Such a rigid binding mode of P3V8 is further supported by the observation that tandem repeats of the peptide failed to interact with the expanded RNA. Studies on the conformation and dynamics of P3V8 before and after RNA binding are needed to understand the mechanism of its specific inhibitory activity.

RNAs are becoming more recognized as attractive therapeutic targets because they fold into well-defined secondary and tertiary structures but exhibit a large variety of conformations, which can provide favorable opportunities for specific drug targeting. One interesting feature of the A’ form of *CAG* RNA is the widened major groove caused by the non-canonical AA base pairs^29^. Such widening provides binding sites that are unique to CAG-repeat RNA, and their accessibility may offer opportunity to improve the potency and specificity of our inhibitor. Further structural information on the interaction between P3V8 and *CAG* RNA will be useful to provide insight into how to make use of such a unique feature. Nevertheless, the results of our study demonstrates that neutralization of the N- and C-termini by simple modifications like acetylation and amidation can have significant effects on the binding and inhibitory properties of peptidyl inhibitor against an RNA target.

In the past few decades, several inhibitors targeting protein toxicity^30–33^ or *CAG* RNA toxicity^21,34^ in polyQ disease have been developed. Some even showed therapeutic potential^30–33^. In this study, we demonstrated for the first time a structural activity relationship (SAR) investigation of the peptidylic inhibitor, P3, toward *CAG* RNA toxicity in polyQ diseases. The SAR study led us to identify a more potent peptide derivative of P3, P3V8, which shows dramatically improved inhibitory efficacy against expanded RNA-mediated toxicity *in vivo*. However, many peptides have short half-life *in vivo*, typically in minutes, which raises concern whether P3V8 could be administered as a therapeutic agent. Previous studies have established that covalent anchoring of lipids to peptides promotes peptide binding to the fatty acid binding sites on albumin^35^. As albumin has a very long half-life, this ‘docking’ into albumin can significantly extend the functional half-life of peptides. Furthermore, we speculated that lipidation of a peptide could improve the ability to cross cell membranes and enter cells. We therefore lipidated P3V8 by N-acylation with palmitic acid. Stability studies of the lipidated peptide L1P3V8 in plasma and brain homogenates revealed that lipidation significantly enhanced the cellular uptake and *in vivo* stability of P3V8. Above all, the lipidation strategy notably improved the pharmacokinetic profile and brain uptake of L1P3V8 in rats when it was administered intranasally, conferring our inhibitor drug/lead-like properties. The ability of therapeutic agents to pass the blood-brain-barrier is one of the most critical requirements for treatment of neuronal diseases. Although the mechanism of brain uptake of L1P3V8 remains to be elucidated, our results illustrate that the combination of lipidation and intranasal administration may provide a new means to improve the brain uptake of therapeutic agents. In conclusion, our results provide proof of concept that lipidated peptidyl inhibitors that target RNA toxicity are a novel therapeutic option for polyQ diseases. Further modification and optimization on this lead-like L1P3V8 will be needed to prolong its half-life and uptake level in the brain.

## Methods

### Peptide modeling

Molecular modeling of P3V8 was carried out using the online PEP-FOLD 2.0 server^23^. The sequence of the 13 amino acid peptide was submitted to the server and 100 simulations were performed using the default settings. The program returned the most representation conformations identified in terms of energy and population, and clustered them based on their sOPEP (Optimized Potential for Efficient structure Prediction) coarse grained energies. The representative model of the top-ranked cluster with the lowest sOPEP energy values was selected for this study.

### Construction of plasmids

The pcDNA3.1-MJD_CAG27_, pcDNA3.1-MJD_CAG78_, pcDNA3.1-MJD_CAA/G78_ and pEGFP_CAG78_ constructs were reported previously^7,13^.

### Synthesis of peptides and *CAG* RNAs

P3WT and P3 mutant (MT) peptides were purchased from GenScript USA Inc. All other peptide variants were prepared by Fmoc solid-phase peptide synthesis on automated peptide synthesizers, Biotage Syro Wave and Biotage SP Wave instruments, and assembled on a 0.1 mmol scale, unless noted otherwise. Peptide syntheses were carried out on a TentaGel S Rink Amide resin (0.22 mmol/g). Amino acids had Fmoc protection of Nα-amino groups; side-chain protecting groups were tert-butyl (Tyr, Glu, Asp), 2,2,4,6,7-pentamethyl-dihydrobenzofuran-5-sulfonyl (Pbf, for Arg and hArg), and *tert*-butyloxycarbonyl (Boc, for Lys, Orn and Dap). Amino acids, NMP, DMF and piperidine were supplied by Iris Biotech (Germany). Acetonitrile, formic acid, triethylsilane (TES), trifluoroacetic acid (TFA) acetic anhydride, and dichloromethane (DCM) were from Sigma-Aldrich (Denmark). All chemicals were used as received and without further purification.

Analytical HPLC was performed with a Dionex Ultimate 3000 instrument on a Phenomenex Germini-NX C18 column (3 μm, 50 × 4.6 mm), column oven thermostated to 42 °C, and a linear gradient flow of CH_3_CN-H_2_O (0.1% formic acid), connected to an ESI-MS (MSO Plus Mass Spectrometer, Dionex). Purification of the peptide was performed on a preparative Dionex Ultimate 3000 HPLC with a C18 column Phenomenex Gemini Axia (5 μm, 100 × 21.2 mm, 110 Å). Unless otherwise stated CH_3_CN-H_2_O (0.1% TFA or 0.1% formic acid) was used as eluent with a flow of 15 mL/min. Gradient elution for 0–5 min was 5%, then to 55% for 5–32 min.

All standard Fmoc amino acids were coupled in DMF with 5.2 equivalents of amino acids and HOAt, with 5 equivalents of HBTU and 9.4 equivalents DIEA. The coupling time was 60 min at room temperature. All non-standard amino acids, as well as palmitic acid, were coupled using 2 equivalents of amino acids and HOAt, 1.9 equivalents of HBTU and 3.6 equivalents DIEA. Coupling times were 10 min at 75 °C. N-terminal fatty acid was introduced using the same conditions as for the coupling of standard amino acids.

In the synthesis of peptides P3V7 to P3V21, the first 10 couplings used a coupling time of 60 min at 25 °C with NMP and washings in-between couplings. Deprotections were performed by treatment with piperidine-DMF (2:3) for 3 min, followed by piperidine-DMF (1:4) for 15 min. After each coupling and deprotection, a washing procedure with NMP (3x), DCM (1x), then NMP (3x) was performed. From the 10th coupling onwards, the coupling time was increased to 2 × 120 min and an extra deprotection step (15 min) with piperidine-DMF (1:4) was added. The N-terminal acetylation was achieved with acetic anhydride in DMF (1:4) for 2 × 15 min. After completion of peptide chain assembly, the resin was washed 6 times with DCM. All the peptides were released and deprotected by treatment with a cocktail of trifluoroacetic acid (TFA), triethylsilane (TES) and H_2_O (95:2:3 or 95:2.5:2.5) for 2 hr. The TFA solutions were concentrated under a flow of nitrogen and the compounds were precipitated with diethylether to yield the crude products. All peptides were purified by RP-HPLC.

The sequences of peptides are listed in Table 1, Supplementary Tables S1 and S2. The purity of peptides used in cell experiments and *in vitro* binding was over 90%. Desalted peptides were used in *Drosophila* feeding assays. All RNAs were synthesized using the MEGAscript^®^ kit (Ambion) as previously described^13^, and the *MJD*_*CAG27*_, *MJD*_*CAG78*_ and *MJD*_*CAA/G78*_ RNAs were transcribed from linearized *pcDNA3*.*1-MJD*_*CAG*_ constructs^36^.

### Isothermal titration calorimetry binding assay

Experiments were carried out using a MicroCal iTC200 isothermal titration calorimeter (GE Healthcare) at 25 °C. Data were analyzed using the Origin^®^ scientific plotting software version 7 (Microcal Software Inc.). All RNAs and peptides were dissolved in binding buffer (20 mM MOPS, pH 7.0; 300 mM NaCl) and 0.7 mM of peptide was titrated into 0.5 µM of RNA for each experiment. The concentration of RNA was estimated with appropriate extinction coefficients at 260 nm on a Nanodrop 2000 (Thermo Scientific). A reference power of 8 μcal/s was used with an initial 0.5 µl of injection of peptide followed by 2 µl for all subsequent titrations points with a 60 sec initial equilibrium delay and 150 sec pause between injections. The samples were stirred at a speed of 1000 rpm throughout the experiment. The thermal titration data were fitted to the ‘one binding site model’ to determine the dissociation constant (K_D_). Each experiment was repeated at least 3 times with consistent results obtained.

### Cell culture, plasmid transfection and peptide transfection

HEK293 cells were cultured at 37 °C with 5% CO_2_ in DMEM supplemented with 10% FBS and 1% penicillin-streptomycin. Primary rat cortical neurons were isolated and cultured as previously described^37^. Transient transfection of HEK293 cells was performed using Lipofectamine 2000 (Life Technologies). P3V8 were delivered to HEK293 cells using the DeliverX (DX) Peptide Transfection Kit (Affymetrix) 4 hr after DNA transfection. At least two batches of independently synthesized peptides were used in the experiments.

### Lactate dehydrogenase (LDH) cytotoxicity assay and IC_50_ determination

To detect the effect of P3V8 and lipidated P3V8 (L1P3V8) on inhibiting cell death in *EGFP*_*CAG78*_ RNA-expressing HEK293 cells, a LDH assay was employed. HEK 293 cells were seeded on a 24-well plate at a density of 0.5 × 10^5^, and *pEGFP*_*CAG78*_ DNA construct was used to transfect the cells. For P3V8 treatment, different concentrations of P3V8 as indicated in the figure were transfected by DeliverX transfectant 4 hr after DNA transfection. For L1P3V8 treatment, different concentrations of L1P3V8 were added into individual wells immediately after DNA transfection. Seventy-two hours after treatment, LDH enzyme activity in the cell culture medium was measured as described before. Experimental groups were normalized to the untransfected control. After normalization, data were analyzed using the dose response-inhibition curve (nonlinear regression-variable slope) to determine the IC_50_ value (Prism6 software, GraphPad Software, Inc.). Each experiment was repeated at least 3 times.

### *Drosophila* genetics, peptide feeding and assays

Flies were raised at 21.5 °C on cornmeal medium supplemented with dry yeast. Fly lines bearing *UAS-DsRed*_*CAG0*_ and *UAS-DsRed*_*CAG100*_ were kind gifts of Professor Nancy Bonini (University of Pennsylvania, USA). The *gmr-GAL4* fly line was obtained from Bloomington *Drosophila* Stock Center. For pseudopupil assay, third instar larvae were fed with various amount of respective peptides dissolved in 2% sucrose solution for 2 hr and then continued to culture in standard fly food at 21.5 °C^38^. Pseudopupil assay was performed on 12 day-old adult flies as mentioned previously^39^. Images were captured by SPOT Insight CCD camera controlled by the SPOT Advanced software (Diagnostic instruments Inc.). Image processing was performed using the Adobe Photoshop CS software (Adobe). Each experiment was repeated at least 3 times (n = 10 fly heads), and consistent results were obtained. For viability test of wild-type flies from egg to adult stage, eggs born within 5 hr were collected and cultured in fly food containing 10 or 50 μM of respective peptides at 21.5°C. Viability from egg-to-adult was calculated as the number of adult flies collected divided by the number of eggs examined. Each experiment was repeated for three times (at least 130 eggs were examined in each group). Two batches of independently synthesized peptides were used in the experiments.

### RNA extraction, reverse transcription-PCR and real-time PCR

RNA was extracted from cells or ten 12 day-old adult fly heads by Trizol reagent (Life Technologies), and 1 μg of purified RNA was then used for reverse-transcription using the ImPromII™ Reverse Transcription System (Promega). Random hexamer (Roche) was used as primers in reverse transcription. Taqman gene expression assays were performed on an ABI 7500 Real-time PCR system and data were analyzed as previously described^13^. The following probes were used: *pre-45s rRNA* (Assay ID: AILJIZM), *pre-rRNA* (Assay ID: AIMSG5U), *Drosophila GAPDH* (Assay ID: Dm01841186) and human *actin* (Assay ID: Hs99999903_m1). Each experiment was repeated at least 3 times.

### *In vitro* stability studies in rat plasma and brain homogenate

Sprague-Dawley (SD) rats (male, 180–200 g) were supplied by the Laboratory Animal Services Centre at The Chinese University of Hong Kong. All animal studies were conducted under the approval of the Animal Ethics Committee of The Chinese University of Hong Kong, and were performed in accordance with relevant guidelines and regulations. Blank rat brain homogenate was prepared by ultrasonic probe homogenization (Micoson XL-2000, Misonix, Framingdale, NY, USA) of brains collected from the control rats. Blank rat plasma was prepared by centrifugation (8000 rcf for 3.5 min) of blood collected from control rats. For the stability test, 2000 ng/mL and 500 ng/mL of P3V8 or L1P3V8 was spiked into the blank rat plasma or brain homogenate and vortexed. The mixtures were incubated at 37 °C with 100 rpm in a water bath for 1 or 3 hr. The incubation was terminated by addition twice the volume of acetonitrile and centrifugation at 13000 rcf for 10 min. The supernatant was collected for LC/MS/MS analysis.

### Cellular uptake studies in HEK293 cells

HEK293 cells were seeded on a 6-well plate at a density of 6 × 10^5^ and cultured overnight at 37 °C with 5% CO_2_ in DMEM supplemented with 10% FBS and 1% penicillin-streptomycin. Cells were then treated with 100, 500 or 1000 nM of respective peptide at 37 °C for 3 hr. After treatment, the cells were washed with ice-cold PBS and then lysed by 2% SDS solution. Aliquot (100 µl) of the cell lysate was added with 200 µl acetonitrile and centrifuged at 13000 rcf for 10 min to precipitate the proteins. The collected supernatant was subjected to LC/MS analysis. The protein concentration of the cell lysate was measured using a bicinchoninic acid protein assay kit following Sigma’s protocol.

### Plasma pharmacokinetic and brain uptake studies in SD rat

SD rats (180–200 g) were anesthetized with an intraperitoneal injection of ketamine (60 mg/kg) and xylazine (6 mg/kg) and received a minor surgery of cannulation with a polythene tube (0.4 mm i.d., 0.8 mm o.d., Harvard Apparatus, Holliston, MA, USA) in the left jugular vein. The rats received an overnight recovery with free access to food and water. In the following day, rats were administered P3V8 or L1P3V8 (3 μmol/kg) via intravenous or intranasal routes. For intravenous administration, appropriate volume of P3V8 or L1P3V8 (3 μmol/mL, dissolved in water) was injected to the rats via the cannula. For intranasal administration, the rats were temporarily anesthetized by inhalation of carbon dioxide, and was administrated with appropriate volume of P3V8 or L1P3V8 (30 μmol/mL, dissolved in water with 5% PEG400) in both nostrils by a micropipette. Chitosan solution (0.5% w/v, pH 6.5, 20 μL) was pre-treated to rats intranasally 5 min before the P3V8 and L1P3V8 was administrated via intranasal routes. For the pharmacokinetic study, after the drug administration, blood samples were collected from the catheter at appropriate time intervals (2, 5, 10, 15, 20, 30, 45, 60, and 90 min). After each collection, 0.2 mL of saline containing 25 IU heparin was injected to compensate for the blood loss. Plasma was collected after centrifugation at 8000 rcf for 3.5 min and stored at −80°C until analysis. For brain uptake study, at 20 min, the rat was anesthetized and perfused by 500 mL saline, and the whole brain was collected. The brain was wiped by tissue paper to remove excess water, meninges and blood vessels followed by storage at −80 °C until analysis.

### Sample preparation for LC/MS/MS

Aliquot of plasma sample (80 μL) was mixed with 160 μL acetonitrile. The mixture was vortexed for 1 min and followed by centrifugation at 13000 rcf for 10 min. The supernatant was collect and subjected to LC/MS/MS analysis.

To prepare brain homogenate, the whole brain of each rat was minced. Saline (2 mL/g brain) was added followed by ultrasonic probe homogenization on ice. For analysis of P3V8, aliquot of brain homogenate (100 μL) was mixed with 200 μL acetonitrile. The mixture was vortexed for 1 min and followed by centrifugation at 13000 rcf for 10 min to collect the supernatant. For analysis of L1P3V8, aliquot of brain homogenate (650 μL) was centrifuged at 6000 rcf for 10 min. After the supernatant was collected, 400 μL saline was added to re-suspend the pellet. The mixture was again vortexed for 5 min and centrifuged at 6000 rcf for 10 min. The supernatant collected was loaded to a preconditioned Oasis® HLB cartridges (Waters, MA, USA). After washed with 1 mL of 10% acetonitrile in water, the analyte was eluted with 0.25 mL 95% acetonitrile in water.

### LC/MS/MS analysis

Agilent 6430 Triple Quadrupole LC/MS/MS system (Agilent Technologies, CA, USA) was employed for the analysis. For analysis of P3V8, chromatographic separation was achieved on a SunFire C8 Column (250 mm × 4.6 mm, 5 µm). The mobile phase of water containing 0.2% formic acid (A) and acetonitrile (B) was used with a gradient elution (0–5 min, 20–70% B). The flow rate was 0.8 mL/min. Multiple reactions monitoring (MRM) with fragmentation transition of 500 to 129 in positive ion mode was employed for quantization of P3V8. For analysis of L1P3V8, Alltima Amino Alltech Column (250 mm × 4.6 mm, 5 µm) was used for separation. Mobile phase of water (A) and acetonitrile (B) was used. The gradient elution for plasma samples was 0–4 min, 30–95% B, and for brain samples was 0–9 min, 10–95% B. The flow rate was 0.8 mL/min. MRM transition of 565 to 88 in positive ion mode was used for quantization of L1P3V8.

### Data analyses

Plasma concentration verse time profiles were analyzed by WinNonlin (Pharsight Corporation, Mountain View, CA, USA, Version 2.1) to obtain the pharmacokinetics parameters. Other data were analyzed by one-way ANOVA followed by *post hoc* Tukey test or unpaired t-test. “*”, “**”, “***” and “****” represent *P* < 0.05, *P* < 0.01, *P* < 0.001 and *P* < 0.0001 respectively, which are considered statistically significant.

## Electronic supplementary material

Supplementary information
