# Supplementary material for: A brain-targeting lipidated peptide for neutralizing RNA-mediated toxicity in Polyglutamine Diseases
Source: Sci Rep. 2017 Sep 21;7:12077. doi: 10.1038/s41598-017-11695-y (PMC5608758; doi:10.1038/s41598-017-11695-y)
Supplement: Supplementary file 1 — Supplementary information [file 41598_2017_11695_MOESM1_ESM.pdf]

A brain-targeting lipidated peptide for neutralizing RNA-mediated toxicity in Polyglutamine Diseases

Qian Zhang[a], Mengbi Yang[b1], Kasper K. Sørensen[c1], Charlotte S. Madsen[c], Josephine T. Boesen[c], Ying An[a], Shao Hong Peng[a], Yuming Wei[a], Qianwen Wang[b], Knud J. Jensen[c], Zhong Joan Zuo[b], Ho Yin Edwin Chan[a,d*], Jacky Chi Ki Ngo[a*]

[a]School of Life Sciences, The Chinese University of Hong Kong, Shatin, Hong Kong SAR, China

[b]School of Pharmacy, The Chinese University of Hong Kong, Shatin, Hong Kong SAR, China

[c]Department of Chemistry, University of Copenhagen, Thorvaldsensvej 40, 1871 Frederiksberg, Denmark

[d]Gerald Choa Neuroscience Centre, The Chinese University of Hong Kong, Shatin, Hong Kong SAR, China

[1]These authors contributed equally to this work

***Corresponding authors:**

Ho Yin Edwin Chan

Email: hyechan@cuhk.edu.hk

Phone: +852 3943 4021

Jacky Chi Ki Ngo

Email: jackyngo@cuhk.edu.hk

Phone: +852 3943 6346

Tables

Supplementary Table S1. Binding affinity of P3WT and P3 mutants toward expanded *MJD_{CAG78}* RNA

Peptide	Sequence	K_D (μM)
P3WT	Asp-Gly-Lys-Ser-Lys-Gly-Ile-Ala-Tyr-Ile-Glu-Phe-Lys	8.37±3.83
P3MT1	Asp-Gly-Ala-Ser-Lys-Gly-Ile-Ala-Tyr-Ile-Glu-Phe-Lys	31.27±8.29
P3MT2	Asp-Gly-Lys-Ser-Ala-Gly-Ile-Ala-Tyr-Ile-Glu-Phe-Lys	100.60±13.99
P3MT3	Asp-Gly-Lys-Ser-Lys-Gly-Ile-Ala-Ala-Ile-Glu-Phe-Lys	16.61±2.83
P3MT4	Asp-Gly-Lys-Ser-Lys-Gly-Ile-Ala-Tyr-Ile-Glu-Ala-Lys	17.38±5.19
P3MT5	Asp-Gly-Lys-Ser-Lys-Gly-Ile-Ala-Tyr-Ile-Glu-Phe-Ala	51.30±13.12

P3WT or P3 mutants (0.7 mM) were titrated into *MJD_{CAG78}* RNA (0.5 μ M) and the thermal titration data were fitted to the 'one binding site model' to determine the dissociation constant (K_D). WT indicates wild type and MT indicates mutant. Data are expressed as mean ± S.E.M. for at least 3 independent experiments.

Supplementary Table S2. Binding affinity of P3V14-21 toward expanded *MJD_{CAG78}* RNA

Peptide	Sequence	K_D (μM)
P3WT	Asp-Gly-Lys-Ser-Lys-Gly-Ile-Ala-Tyr-Ile-Glu-Phe-Lys	8.37±3.83
P3 variant 14 (P3V14)	Ac-Asp-Gly-(hArg)-Ser-Lys-Gly-Ile-Ala-Tyr-Ile-Glu-Phe-Lys-NH_2	No binding
P3 variant 15 (P3V15)	Ac-Asp-Gly-Lys-Ser-(hArg)-Gly-Ile-Ala-Tyr-Ile-Glu-Phe-Lys-NH_2	No binding
P3 variant 16 (P3V16)	Ac-Asp-Gly-(Orn)-Ser-Lys-Gly-Ile-Ala-Tyr-Ile-Glu-Phe-Lys-NH_2	No binding
P3 variant 17 (P3V17)	Ac-Asp-Gly-Lys-Ser-(Orn)-Gly-Ile-Ala-Tyr-Ile-Glu-Phe-Lys-NH_2	No binding
P3 variant 18 (P3V18)	Ac-Asp-Gly-Asp-(Dap)-Ser-Lys-Gly-Ile-Ala-Tyr-Ile-Glu-Phe-Lys- NH_2	No binding
P3 variant 19 (P3V19)	Ac-Asp-Gly-Asp-Lys-Ser-(Dap)-Gly-Ile-Ala-Tyr-Ile-Glu-Phe-Lys- NH_2	No binding
P3 variant 20 (P3V20)	Ac-Asp-Gly-Lys-Ser-Lys-Gly-Ile-Ala-Tyr-Ile-Glu-Phe-Lys-Asp-Gly-Lys-Ser-Lys-Gly-Ile-Ala-Tyr-Ile-Glu-Phe-Lys-NH_2	No binding
P3 variant 21 (P3V21)	Ac-Asp-Gly-Lys-Ser-Lys-Gly-Ile-Ala-Tyr-Ile-Glu-Phe-Lys-Gly-Asp-Gly-Lys-Ser-Lys-Gly-Ile-Ala-Tyr-Ile-Glu-Phe-Lys-NH_2	No binding

P3WT or P3 variants (0.7 mM) were titrated into *MJD_{CAG78}* RNA (0.5 μM) and the thermal titration data were fitted to the 'one binding site model' to determine the dissociation constant (K_D). WT indicates wild type. Data are expressed as mean ± S.E.M. for at least 3 independent experiments.

Supplementary Table S3. Binding affinity of P3V8 toward *MJD*$_{CAG27}$, *MJD*$_{CAG78}$ and *MJD*$_{CAA/G78}$ RNA

Peptide	RNA	K_D (μM)
P3V8	*MJD*$_{CAG27}$ RNA	2.19±0.27
P3V8	*MJD*$_{CAG78}$ RNA	0.33±0.04
P3V8	*MJD*$_{CAA/G78}$ RNA	2.94±0.96

P3V8 (0.7 mM) was titrated into *MJD*$_{CAG27/78}$ RNA or *MJD*$_{CAA/G78}$ RNA (0.5 μM). The thermal titration data were fitted to the 'one binding site model' to determine the dissociation constant (K_D). Data are expressed as mean ± S.E.M. for at least 3 independent experiments.

Figures and Figure Legends

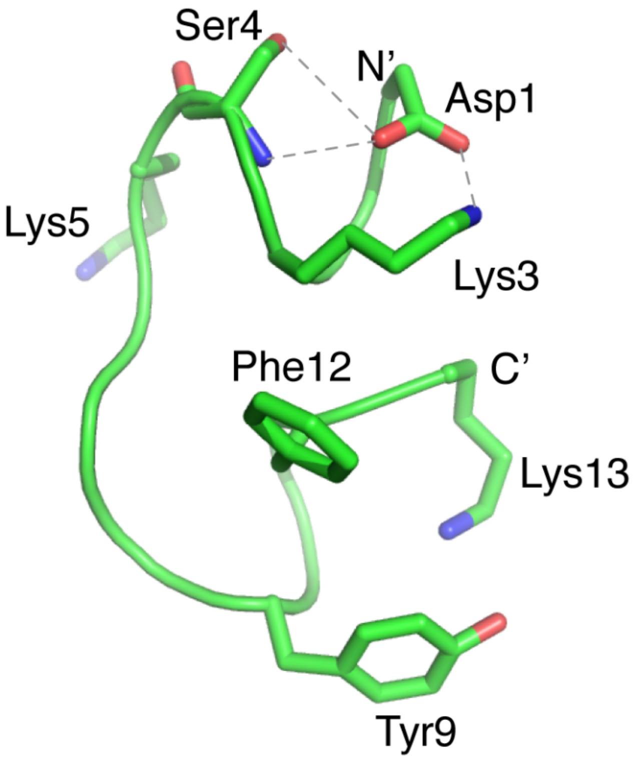

Supplementary Figure S1. Predicted fold of P3V8 peptide. 3D structure of P3V8 predicted by PEP-FOLD[23], where the peptide preferentially adopts a coil conformation stabilized by a network of hydrogen bonds mediated by the side chain of Asp1. The figure was prepared using PyMol[40].

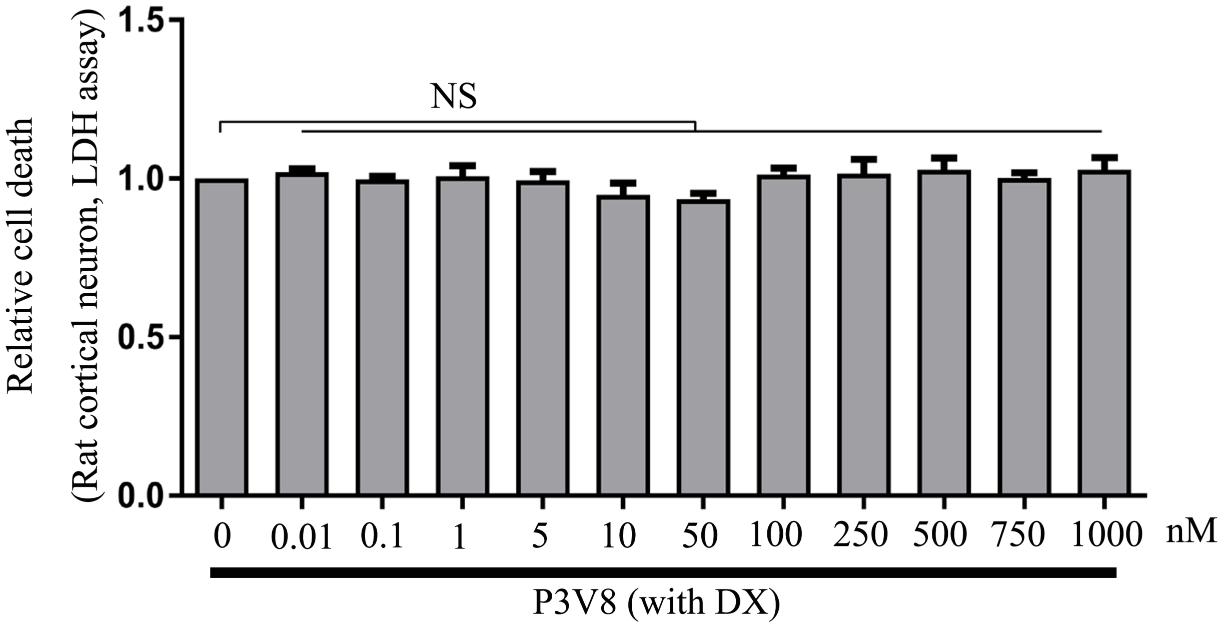

Supplementary Figure S2. Treatment of P3V8 causes no cytotoxicity in primary rat cortical neurons. A density of 5 x 10^5 of primary rat cortical neurons were seeded on culture dish and individually cultured in medium containing 0.01, 0.1, 1, 5, 10, 50, 100, 250, 500, 750, and 1000 nM of P3V8 peptide (transfected with DeliverX). The lactate dehydrogenase (LDH) cytotoxicity activity was measured 72 hr post-treatment. Fold change of LDH activity was normalized to untreated cells. Data are expressed as mean ± S.E.M. for 3 independent experiments. NS indicates no significance.

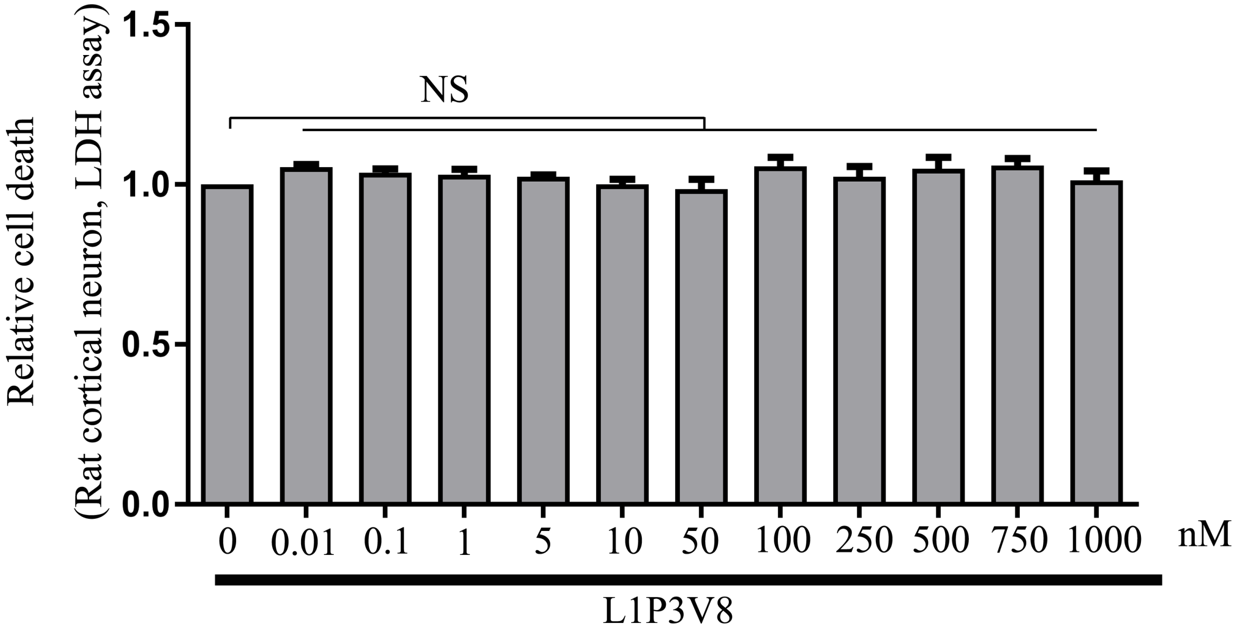

Supplementary Figure S3. Treatment of L1P3V8 causes no cytotoxicity in primary rat cortical neurons. A density of 5 x 10^5 of primary rat cortical neurons were seeded on culture dish and individually cultured in medium containing 0.01, 0.1, 1, 5, 10, 50, 100, 250, 500, 750, and 1000 nM of L1P3V8 peptide. The LDH cytotoxicity activity was measured 72 hr post-treatment. Fold change of LDH activity was normalized to untreated cells. Data are expressed as mean ± S.E.M. for 3 independent experiments. NS indicates no significance.

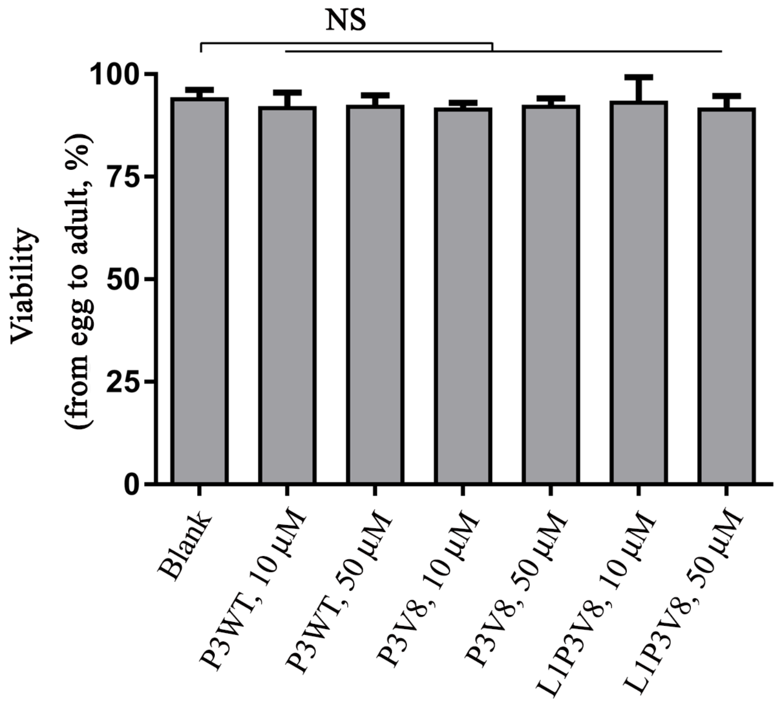

Supplementary Figure S4. Treatment of P3WT, P3V8 and L1P3V8 did not affect the viability of wild type *Drosophila* from egg to adult. Eggs laid within 5 hr were collected and cultured in fly food containing 10 or 50 μM of respective peptides at 21.5°C. Viability from egg to adult was calculated as the number of adult flies collected divided by the number of eggs examined. Data are expressed as mean ± S.E.M. for 3 independent experiments. NS indicates no significance.

Reference

40. Schrodinger, L. L. C. The PyMOL Molecular Graphics System, Version 1.8. http://www.pymol.org (2015).
